# An Ignored Population in Emergency Department: Cardio-Oncology Patients

**DOI:** 10.3390/life15040608

**Published:** 2025-04-06

**Authors:** Ömer Salt, Cafer Zorkun, Semra Aytürk Salt

**Affiliations:** 1 Department of Emergency Medicine, Kayseri City Training and Research Hospital, Kayseri 38060, Turkey; 2 Department of Cardiology, Faculty of Medicine, Istanbul University, Istanbul 34093, Turkey; zorkun@istanbul.edu.tr; 3 Department of Endocrionology and Metabolism Diseases, Kayseri City Training and Research Hospital, Kayseri 38060, Turkey; s.ayturk@hotmail.com

**Keywords:** cardiology, emergency, oncology

## Abstract

**Background:** The aim of this study was to analyze cancer patients who were admitted to the emergency department with cardiac symptoms and hospitalized in the cardiology service or cardiology intensive care unit. **Methods:** One hundred and thirty-one cancer patients who were hospitalized in the period of 5 years were included in the study. Age, sex, type of cancer, treatment, emergency department diagnosis, laboratory parameters, and in-hospital outcomes were evaluated. **Results:** The most common hospitalization diagnosis was acute coronary syndromes (69.5%, *n* = 91). The mortality rate was 14.5% (*n* = 19). The NTproBNP levels were found to be higher in all patients, and especially high in patients with LVEF < 40%. **Conclusions:** Cancer patients with low LVEF and elevated NTproBNP levels and increased HEART and TIMI scores have increased risk for cardiac toxicity and mortality. This patient group should be treated and followed-up with great care.

## 1. Introduction

Cardiovascular diseases are the leading cause of death in our country as well as globally, with cancer-related deaths taking second place [1,2]. In addition, the survival rates of these patients increase in parallel with developments in cancer treatment [3]. Chest pain is the one of the most common complaints of admission to the emergency department [4]. Another important sustained cardiac problem is arrhythmia, especially atrial fibrillation and flutter [5,6]. However, this could be different in the patient population on cancer treatment due to the nature of chemotherapeutics. There is also an interesting role of emergency departments in the diagnosis of cancer, which are generally late-stage cancers [7]. Many patients may hesitate to go to hospitals because they cannot fully evaluate the symptoms or because they are afraid of being diagnosed with a bad disease. As a result, patients may present to emergency services with much more severe symptoms and may be diagnosed at an advanced stage as their disease progresses. This situation particularly results in cancer patients being diagnosed in advanced stages in emergency rooms. There are different biomarkers and scoring systems in the diagnosis and risk assessment of cardiac pathologies. However, the most widely used are hsTroponin, NTproBNP, HEART (history, ECG, age, risk factors, and troponin level), and TIMI (thrombolysis in myocardial infarction) scores [4]. Another problem for these patients is overcrowded emergency departments, which may result in this patient population not receiving adequate service [8]. Patients receiving cancer treatment are at increased risk in terms of cardiovascular complications compared with other oncologic patients because of the adverse effects of chemotherapeutic agents and radiotherapy used in the treatment [9,10]. In light of all these factors, there is a need for the formation of a subspecialty called cardio-oncology to reduce the cardiac risks of patients undergoing cancer treatment [11]. These clinics mainly serve on the following parameters: determination of the patient’s cardiac status before cancer treatment, minimization of the cardiotoxic effects during therapy, the development of personalized cardiac protection programs, and the establishment of appropriate follow-up strategies [12]. The first cardio-oncology clinic of our country was put into service in May 2016 for the purpose of the cardiac follow-up of patients receiving cancer treatment. In this clinic, the pre-treatment cardiovascular risk assessment of patients as well as preventive treatment regimens, cardiovascular risk reduction in patients, and support treatments with medium- and long-term follow-up are performed. The aim of this study was to analyze patients who were admitted to the emergency department with cardiac symptoms and determine the cardiac markers and scoring systems for the diagnosis in the period of five years and hospitalized in the cardiology service or cardiology intensive care unit.

## 2. Materials and Methods

The study was conducted at Trakya University Faculty of Medicine Research Hospital, Edirne/Turkey, which is a third level training-research hospital. Following the approval of the ethics committee (Scientific Researches Ethical Committee, Reference Number: TUTF-BAEK 2018/221), a total of 302 patients who were admitted to the ED with cardiac symptoms and transferred to the cardiology unit were detected in the study period. However, 171 of them were excluded from the study due to having a previous history of cardiac disease, comorbid diseases such as chronical kidney failure, chronic obstructive pulmonary disease, etc., or having no echocardiographic evaluation and NTproBNP levels before cancer treatment.

A total of 131 patients who were admitted to the emergency department with cardiac symptoms or diagnosed as having a cardiac pathology and then transferred to the cardiology service or cardiology intensive care in the period of 5 years were included in the study. All patients were previously healthy and had no comorbid disease. They were all examined in terms of cardiac pathologies and had echocardiographic evaluation, hsTroponin, NTproBNP levels, HEART, and TIMI scores before chemotherapy. Throughout the study, echocardiographic evaluations of all patients were performed by the same cardio-oncologist who was an expert in this field. Our hospital’s cardio-oncology clinic works in coordination with the oncology clinic. Therefore, the first echocardiographic evaluations of patients are performed before the oncological treatment is started. Control echocardiography of patients who applied to the cardio-oncology outpatient clinic during follow-up was also performed by the same cardio-oncologist. In this way, we aimed to make the follow-ups more reliable. Echocardiographic examination was performed in accordance with the EACVI/American Society of Echocardiography (ASE) recommendations. The age, gender, type of cancer, treatment, emergency department diagnosis, laboratory parameters, and the mortality status of patients who were followed-up in both the cardiology intensive care unit and the cardiology ward during the oncological treatment period were also recorded in the previously created study form (Supplementary Materials). Among the patients who had repeated emergency department visits during the study period, only those whose admissions were their first hospital stay were included in the study. Before initiating cancer treatment, all patients underwent echocardiographic evaluation at the cardio-oncology outpatient clinic. Only patients with pre-oncological treatment of the left ventricular ejection fraction (LVEF) above 40% were included in the study. All patients included in the study had normal pre-treatment hsTroponin I and NTproBNP levels. The LVEF, hsTroponin, and NTproBNP levels, along with the TIMI and HEART scores, were reassessed following the patients’ hospital admission. Statistical analyses were performed to compare other parameters between these two groups.

The data were recorded using the SPSS statistical program and statistical analysis was performed. Statistical evaluation was performed by using IBM SPSS Statistics for Windows, Version 21.0 (Armonk, NY, USA, IBM Corp.). The Shapiro–Wilk test was used for the assessment of normal distribution. The relationships between qualitative variables were investigated using Pearson’s Chi-square test and Fisher’s exact test (if the expected value of at least one of the eyes in the 2 × 2 tables was below 5 and the expected value of at least 20% of the eyes in the multi-eyed tables was below 5). The dependent samples *t*-test was used to compare the sample means from two related groups. Categorical variables were compared using the Chi-square test, while continuous variables with normal distribution were analyzed using the independent *t*-test, and non-normally distributed variables were analyzed using the Mann–Whitney U test. The Mann–Whitney U test was applied to compare the hsTroponin I and NTproBNP levels between the LVEF < 40% and LVEF > 40% groups. For descriptive statistics, quantitative variables were given as the median, smallest, and maximum value, and qualitative variables were given as number and percentage. The significance level was determined as 0.05 in all statistical analyses.

## 3. Results

Of the patients included in the study, 27.5% (*n* = 36) were female and 72.5% (*n* = 95) were male. The mean age was 68.4 ± 11.5 years. The demographic characteristics, final diagnoses, and cancer type of the patients are shown in Table 1.

Before initiating cancer treatment, the left ventricular ejection fraction (LVEF) rate of all patients was higher than 40% (51 ± 6.3% (mean ± SD)) and they had normal NTproBNP levels (<125 pg/mL) (74 ± 21.8 pg/mL (mean ± SD). To determine the cardiac risks of oncological patients before treatment in our clinic, HEART and TIMI scores were calculated and recorded at the time of their first application. The aim here was to determine whether cardiac events related to treatment could be predicted with these scoring systems. While the HEART score of all patients was below 3 before the start of treatment, the HEART scores calculated at the time of emergency service admission were 5 and above. A statistically significant difference was detected in this respect (*p* < 0.01). Similarly, while the TIMI scores of the patients calculated before oncological treatment were all below 3, the TIMI scores calculated at the emergency department admissions were 4 and above. In this respect, a statistically significant difference was found between the TIMI scores at the beginning of treatment and the emergency department admissions (*p* < 0.01). This finding suggests that the patients experienced a significant increase in their HEART and TIMI scores in relation to the treatment.

The most common admission complaints of the patients were not feeling well (42.7%, *n*: 56), shortness of breath (24.4%, *n*: 32), palpitation (13%, *n*: 17). The most common cancer type was lung cancer with *n*: 47 (35.9%). This was followed by prostate cancer with *n*: 25 (19.1%) and breast cancer with *n*: 20 (15.3%). When the cancer types were evaluated among themselves, lung cancer was statistically significantly higher compared with the other cancer types. The cancer types of the patients are shown in Table 2.

When the admission electrocardiograms (ECGs) of the patients were evaluated, it was seen that 49 (37.4%) had ST segment elevated myocardial infarction (STEMI), 47 (35.9%) had atrial fibrillation, 19 (9.9%) were normal sinus rhythm, 12 (9.2%) had sinus tachycardia, 7 (5.3%) had a left bundle branch block, 3 (2.3%) had a right bundle branch block, and 2 (1.5%) patients had ventricular fibrillation. These two patients were defibrillated, and ventricular fibrillation terminated with the return of spontaneous circulation (ROSC). Electrocardiogram rhythms were evaluated statistically among themselves, and STEMI and atrial fibrillation were found to be significantly more frequent than the other pathological ECG changes.

When the hospitalization diagnoses of the patients were examined, 49 (37.4%) patients had acute ST elevated myocardial infarction, 30 (22.9%) had decompensated heart failure, 30 (22.9%) had non-ST-elevation myocardial infarction (NSTEMI), 12 (9.2%) had unstable angina pectoris, 6 (4.6%) had pericardial effusion without pericardial tamponade, 3 (2.3%) had symptomatic bradycardia, and 1 (0.8%) patient had treatment-resistant supraventricular tachycardia. When the patients were evaluated statistically in terms of their hospitalization diagnoses, it was seen that the number of patients who were hospitalized with diagnoses of acute myocardial infarction was significantly higher than the other patient types.

When the patients were evaluated in terms of the type of chemotherapeutic agent they received, it was found that alkylating agents were used most frequently (*n* = 23, 27.7%), followed by monoclonal antibodies (*n* = 16, 19.2%). There was a statistically significant difference in the frequency of alkylating agent use compared with other treatments. Patients receiving chemotherapeutic treatment and their treatment regimens are shown in Table 3.

The mean N-terminal pro B-type natriuretic peptide (NTproBNP) levels of these patients were 3570.93 ± 1062.01, and these values were much higher than the upper limit of normal values. The mean high-sensitivity cardiac troponin assay (hsTroponin I) levels of the patients were 160.21 ± 129.22. When the serum electrolyte parameters of the patients were examined, no major electrolyte disturbance was found. These values are shown in Table 4.

The LVEF ≥ 40% group included 105 patients (80.2%), while the LVEF < 40% group comprised 26 patients (19.8%). Nineteen of these patients (14.5%) died while continuing their treatment after hospital admission. Only intrahospital mortality rates were evaluated, and this period was no longer than one month. The mortality rate was 26.9% (*n* = 7) in the LVEF < 40% group and 11.4% (*n* = 12) in the LVEF ≥ 40% group. When evaluated in terms of mortality rate, the proportion of patients in the LVEF < 40% group was higher, but there was no statistically significant difference between the two groups (*p*: 0.061). Additionally, the hsTroponin I (441.32 ± 134.53) and NTproBNP (5716.56 ± 2413.63) levels in the LVEF < 40% group were significantly higher compared with those in the LVEF ≥ 40% group (114.97 ± 34.43 and 3475.34 ± 1347.93, respectively). However, there was no statistically significant difference between the two groups in terms of the serum electrolyte levels. When the relationship between the type of chemotherapeutic treatment and mortality was evaluated, the highest mortality rate was 50% (*n* = 5) in the group receiving the antimicrotubular agent.

## 4. Discussion

In parallel with the increase in the number of cancer cases worldwide, the number of patients with cancer in our country is increasing day by day. These patients have an increased risk of cardiotoxicity as well as suppression of their immune system. Therefore, cardiac follow-up of these patients is vital. For this purpose, the number of cardio-oncology clinics is increasing. The first of these clinics in our country was established at our university and has been conducting cardiac follow-up and treatment of oncology patients for more than two years. Many risk factors have been defined for the development of treatment-induced cardiotoxicity in these patients. One of the risk factors for cardiotoxicity in patients with cancer has been reported as female sex [13]. However, in our study, only one-quarter of the patients were female patients. We think that the higher number of male patients in our study was accounted for by the high frequency of lung and prostate cancers in our region. As another risk factor, hypertension was found in 75.6% (*n* = 99) of our patients, and such a high rate indicates the importance of hypertension control in these patients.

Another important result of our study was that the in-hospital mortality rate of the patients was 14.5% (*n* = 19), and the diagnoses of all these patients were acute myocardial infarction and ventricular fibrillation. The in-hospital mortality rate is reported to be below 7% in the normal population with acute myocardial infarction [14,15]. We assumed that both treatment-related complications and comorbid conditions are effective in the oncologic patient population, and this ratio is twice as high. From this point of view, we once again emphasize the importance of cardiac evaluation and follow-up in this patient population.

Previous studies have shown that the most important parameter in the cardiac follow-up of oncology patients is the protection of LVEF [16]. In the study by Albini et al., it was reported that there was reduction a between 5 and 20% in the LVEF of the patients who were on chemotherapy. These drugs can directly or indirectly affect the cardiovascular system via thrombogenic status and blood-flow alterations. In our study, the most effective factor on in-hospital mortality was found to be low LVEF in the cancer patient population. Accordingly, it is evident that very close follow-up of the systolic functions of patients who have myocardial toxicity due to cancer treatment is very important in terms of patient health.

Pareek et al. [12] reported that the BNP levels were an early indicator of cardiotoxicity in patients receiving cancer treatment. In our study, the NTproBNP levels were found to be higher in all patients, but especially so in patients with LVEF < 40%. Moreover, these levels were measured during the emergency service admissions of all patients. This was also true for patients who died during hospitalization. Therefore, we think that the NTproBNP levels could be useful as a predictor of in-hospital mortality risk in these patients. In many studies investigating the risk of coronary artery disease in patients receiving chemotherapy [16,17], it has been shown that these patients have an increased risk of coronary artery disease. In our study, 67.2% (*n* = 88) of patients had a previous history of coronary artery disease and only six patients had newly developed coronary artery disease. No statistical evaluation could be made for the chemotherapeutic drugs they received because of the insufficient number of these patients. In this respect, we believe that future studies with more patients will be beneficial.

In a study conducted among patients presenting to the emergency department with chest pain, it was reported that both the HEART and TIMI scores had high predictive value, but the HEART score had a higher predictive value compared with the TIMI score [4]. In our study, both scoring systems were found to have a high predictive value. However, no statistically significant superiority of one scoring system was found over the other. We believe that the presence of comorbid diseases in the patients included in the study by Kagansky et al. [4] may have been effective in this situation because the patients included in our study did not have any comorbid conditions before treatment.

Life-threatening cardiac rhythm disorders can be seen in 16–36% of patients under oncologic treatment [17,18]. However, a low number (5–10%) of these patients are admitted with severe arrhythmias with a need for treatment as inpatients [19]. In our study, pathologic ECG findings were found in 54.2% (*n*: 71) of the patients at the time of admission to the emergency department. However, since we did not have all the patients’ ECG data before starting the treatment, it was not possible to evaluate exactly how much of these pathological rhythms were related to treatment. When the ECG findings of the patients who died were evaluated, supraventricular tachycardia was found to be statistically significant more frequently at 50.4% (*n* = 12) than other pathologic rhythms (*p* = 0.024). In the literature, it is stated that this situation might be related to previous comorbid conditions, left ventricular dysfunction, direct tumor effect, or the toxic effects of cancer treatment [19,20]. When this situation is evaluated from this perspective, it can be seen that patients who have cancer treatment and are admitted with supraventricular tachyarrhythmia have an increased risk of mortality compared with other patients.

In the study by Bossaer et al. [21], it was reported that the most common types of cancer related to cardiotoxicity were multiple myeloma, lung cancer, non-Hodgkin lymphoma, and breast cancer. However, in our study, the frequency ranking was found to be lung, prostate, breast, and stomach cancer, respectively. We think that the type and frequency of cancer in our country was effective in this result. According to the cancer data of 2015, the most common types of cancer in our country were lung, prostate, colon, and bladder, respectively [2]. We believe that this could also be related to the chemotherapy and radiotherapy treatment. It is well-known that there is strong relationship between cardiotoxicity and chemotherapeutic agents such as anthracyclines, antimetabolites, vinca alkaloids, and tyrosine-kinase inhibitors [21]. In our study, we found that the most widely used chemotherapeutic agents were alkylating agents, monoclonal antibodies, and antimicrotubular agents.

Liquid-electrolyte disturbance is another condition that causes cardiac effects in patients undergoing cancer treatment. This may be associated with the underlying malignancy itself or may be associated with the treatment [22]. The most common types of these electrolyte disorders are hyponatremia, hypercalcemia, hypokalemia, and hypomagnesemia [23]. In one study, it was found that 47% of patients who received cancer treatment and were hospitalized for any reason had hyponatremia [24]. In our study, none of the patients had a major electrolyte disorder at admission. Regular assessment of the blood parameters of these patients has great importance in the prevention of electrolyte disorders that may occur during treatment.

One of the most important factors limiting our study was the small number of patients. However, we only included patients with cancer who were admitted to the emergency department and then transferred to the cardiology unit, and the relatively new cardio-oncology unit in this study. Although there were a large number of cardio-oncology patients, we only included the patients who were previously healthy in terms of cardiac condition and had no comorbid disease. This situation limited the number of patients. We also excluded the patients who were treated with radiotherapy because radiotherapy near the cardiac region directly affects the myocardium and LVEF, which could be studied separately in another study.

## 5. Conclusions

We believe that this study may be a guide for emergency department applications of this patient group, especially since the number of studies conducted in the field of cardio-oncology is limited. This descriptive study shows the importance of cardiotoxicity in patients receiving cancer treatment. As a result of this study, it was seen that oncology patients with low LVEF, elevated NTproBNP levels. and increased HEART and TIMI scores had increased risk for cardiac toxicity and mortality. Since this patient population has a high risk of cardiac effects due to the treatments they receive, they need to be evaluated in more detail than the normal population. From this perspective, the importance of cardiac evaluation in this patient group is emphasized once again. We believe that this study will play a guiding role in future studies.

## Figures and Tables

**Table 1:** Demographic characteristics, service diagnoses, and comorbid diseases of the patients.

**Age (Mean ± SD)**	68.4 ± 11.5
Sex	*n* (%)
Male	95 (72.5)
Female	36 (27.5)
Metastasis	48 (36.6)
Oncologic surgery	43 (32.8)
Hospitalized Department	
Cardiology Ward	107 (81.7)
Cardiology ICU **	24 (18.3)
Outcome	
Exitus	19 (14.5)
Discharged	112 (85.5)

** ICU: intensive care unit.

**Table 2:** Oncologic diagnoses of the patients.

Oncologic Diagnosis	*n* %
Lung cancer	47 (35.9)
Prostate cancer	25 (19.1)
Breast cancer	20 (15.3)
Gastric cancer	16 (12.2)
Rectum cancer	8 (6.1)
Colon cancer	4 (3.1)
Liver cancer	3 (2.3)
Pancreatic cancer	2 (1.5)
Endometrial cancer	2 (1.5)
Hematologic malignancy	2 (1.5)
Bladder cancer	1 (0.8)
Skin cancer	1 (0.8)

**Table 3:** Treatment regimens of patients receiving chemotherapy.

Type of Chemotherapeutic	*n* (%)
Alkylating agent	23 (27.7)
Monoclonal antibody	16 (19.2)
Antimicrotubule agent	10 (12.1)
Aromatase inhibitor	9 (10.8)
Immunomodulatory agent	8 (9.6)
Kinase inhibitor	8 (9.6)
Fluoropyrimidine	5 (5.5)
Anthracycline	5 (5.5)

**Table 4:** The mean serum electrolyte level of the patients.

Electrolyte	Serum Level (Mean ± SD)
Sodium (mEq/L)	136.09 ± 4.76
Potassium (mEq/L)	4.37 ± 0.62
Chloride (mEq/L)	103.23 ± 5.44
Calcium (mEq/L)	8.81 ± 0.69
Magnesium (mEq/L)	1.94 ± 0.31

## Data Availability

Data available on request from the authors.

## Supplementary Materials

The following supporting information can be downloaded at: https://www.mdpi.com/article/10.3390/life15040608/s1
