# Supplementary material for: An Ignored Population in Emergency Department: Cardio-Oncology Patients
Source: Life (Basel). 2025 Apr 6;15(4):608. doi: 10.3390/life15040608 (PMC12028992; doi:10.3390/life15040608)
Supplement: Supplementary file 1 [file life-15-00608-s001.zip › life-3537591-supplementary.pdf]

STUDY FORM		
Patient Number:		
Age:		
Gender:		
Oncologic diagnosis:		
Metastasis:	Yes	No
Oncologic surgery	Yes	No
Type of chemotherapeutic		
Serum Sodium at admission of ED	mEq/L	
Serum Potassium at admission of ED	mEq/L	
Serum Chloride at admission of ED	mEq/L	
Serum Sodium at admission of ED	mEq/L	
Serum Calcium at admission of ED	mEq/L	
Serum Magnesium at admission of ED	mEq/L	
Serum Troponin at admission of ED	ng/mL	
Serum NTproBNP at admission to ED	pg/mL	
Left ventricular EF before treatment	%	
Left ventricular EF at admission to ED	%	
Serum NTproBNP before treatment	pg/mL	
HEART score before treatment		
HEART score at admission to ED		
TIMI score before treatment		
TIMI score at admission to ED		
Electrocardiogram at admission to ED		
Emergency Department diagnosis		
Hospitalized department	Cardiology ward	Cardiology ICU
Outcome	Exitus	Discharged
